# Porcine Tissue-Specific Regulatory Networks Derived from Meta-Analysis of the Transcriptome

**DOI:** 10.1371/journal.pone.0046159

**Published:** 2012-09-26

**Authors:** Dafne Pérez-Montarelo, Nicholas J. Hudson, Ana I. Fernández, Yuliaxis Ramayo-Caldas, Brian P. Dalrymple, Antonio Reverter

**Affiliations:** 1 Computational and Systems Biology, Commonwealth Scientific and Industrial Research Organisation (CSIRO) Animal, Food and Health Sciences, Queensland Bioscience Precinct, St. Lucia, Brisbane, Queensland, Australia; 2 Departamento de Mejora Genética Animal, Instituto Nacional de Investigación y Tecnología Agraria y Alimentaria, Madrid, Spain; 3 Departament de Ciència Animal i dels Aliments, Facultat de Veterinària, Universitat Autònoma de Barcelona, Bellaterra, Barcelona, Spain; CRS4, Italy

## Abstract

The processes that drive tissue identity and differentiation remain unclear for most tissue types. So are the gene networks and transcription factors (TF) responsible for the differential structure and function of each particular tissue, and this is particularly true for non model species with incomplete genomic resources. To better understand the regulation of genes responsible for tissue identity in pigs, we have inferred regulatory networks from a meta-analysis of 20 gene expression studies spanning 480 Porcine Affymetrix chips for 134 experimental conditions on 27 distinct tissues. We developed a mixed-model normalization approach with a covariance structure that accommodated the disparity in the origin of the individual studies, and obtained the normalized expression of 12,320 genes across the 27 tissues. Using this resource, we constructed a network, based on the co-expression patterns of 1,072 TF and 1,232 tissue specific genes. The resulting network is consistent with the known biology of tissue development. Within the network, genes clustered by tissue and tissues clustered by site of embryonic origin. These clusters were significantly enriched for genes annotated in key relevant biological processes and confirm gene functions and interactions from the literature. We implemented a Regulatory Impact Factor (RIF) metric to identify the key regulators in skeletal muscle and tissues from the central nervous systems. The normalization of the meta-analysis, the inference of the gene co-expression network and the RIF metric, operated synergistically towards a successful search for tissue-specific regulators. Novel among these findings are evidence suggesting a novel key role of *ERCC3* as a muscle regulator. Together, our results recapitulate the known biology behind tissue specificity and provide new valuable insights in a less studied but valuable model species.

## Introduction

Cell and tissue differentiation proceeds from tightly controlled spatial and temporal patterns of gene expression in the cell. Moreover, differences in gene expression between cell and tissue types are largely determined by transcripts derived from a limited number of tissue-specific (TS) genes, rather than by combinations of more promiscuously expressed genes [1]. Importantly, tissue specificity of gene expression has been associated with different rates of polymorphisms [2], evolution [3], [4], disease association [5] and gene connectivity [6]. The identification of these TS genes is therefore likely to inform and enhance understanding of critical factors contributing to tissue specific function, structure and development. The list of transcriptional regulators driving this process is composed of transcription factors (TF), signaling molecules, co-factors, chromatin remodelers and small RNA molecules, but identifying their role in particular biological processes from expression data remains a challenge [7].

TF interact with each other to regulate the transcriptional output of a gene. However, most existing studies are focused on a limited number of TF. More often than not, it is the synergistic activity of several TF that directs the transcriptional regulation of a particular gene [8]. For this reason, the analysis of all TF interactions in a whole network appears a rational approach to better understand the complete picture of transcriptional regulation. In such a scenario, tissue-specific transcription factors (TSTF) deserve special attention, as they are the key regulators of tissue specific function and differentiation.

Here, in the spirit of meta-analysis approaches frequently invoked in genetic [9] and genomic studies [10], we integrate the data from 20 gene expression studies spanning 480 Porcine Affymetrix chips for 134 experimental conditions on 27 distinct tissues (Table 1). Analogous approaches have been undertaken before in humans, mice, cattle and other species [11], [12]. Resulting from this exercise, herein we compile a matrix comprising the normalized expression of 12,320 porcine genes across 27 tissues.

**Table 1:** Description of the datasets used in this study.

Reference	GEO Acc.	Chips	Tissue(s)*	Brief description
[61]	GSE26701	12	SM	4 postmortem times (20 min, 2 h, 6 h, 24 h) with 3 rep.
[62]	GSE22487	12	LD	4 developmental times (0 d, 7 d, 14 d, 21 d) with 3 rep.
[63]	GSE21383	12	OVA	6 high prolificacy replicates +6 low prolificacy rep.
[64]	GSE19975	6	LD, SOL	2 tissues with 3 rep.
[65]	GSE22165	30	BRAIN	10 conditions (3 treatments* 3/4 times) with 3 rep.
[66]	GSE18641	12	UTE	6 pregnant rep. +6 non-pregnant rep.
[67]	GSE14643	13	HEART	6 untreated rep. +7 treated rep.
[68]	GSE15256	54	ILE	3 conditions* 3 times with 6 rep.
[69]	GSE11853	12	PLA	2 breeds* 2 times with 3 rep.
[70]	GSE11787	6	SPL	3 infected rep.s +3 uninfected rep.
[71]	GSE9333	8	BFT	2 breeds with 4 rep.
[72]	GSE11193	12	LD	6 high drip loss rep. +6 low drip loss rep.
[73]	GSE7314	15	MLN	3 uninfected rep.+(3 infected rep.* 4 times)
[74]	GSE7313	15	MLN	3 uninfected rep. +(3 infected rep.* 4 times)
[75]	GSE10898	64	OLF, HYP, PIN, ADE, NEU, ACO AME, THY, DIA, BIC, BFT, AFT, STO, LIV, ILE, BLO	2 breeds* 16 tissues with 2 rep.
[76]	GSE13528	48	LIV, BFT	2 conditions* 2 genotypes* 2 tissues with 6 rep.
[77]	GSE18359	40	LIV, BFT	2 conditions* 2 RFI levels* 2 tissues with 5 rep.
[78]	GSE21096	20	HEART	4 treatments with 5 rep.
[79]	GSE23596	9	SPL	3 treatments with 3 rep.
[80]	GSE14739	80	HYP, ADE, THY, OVA, TES, BFT	4 breeds* 5 tissues with 4 rep.
TOTAL	20	480	27	

Rep.: replicates.

* Tissue codes are as follows: SM: *Semi-membranosus* muscle; LD: *Longissimus dorsi* muscle; OVA: Ovaries; SOL: *Soleus* muscle; BRAIN: Brain; UTE: Uterus; HEART: Heart; ILE: Ileum; PLA: Placenta; SPL: Spleen; BFT: Back fat tissue; MLN: Mesenteric lymph nodes; OLF: Olfactory bulb; HYP: Hypothalamus; PIN: Pineal gland; ADE: Adenohypophysis; NEU: Neurhypophysis; ACO: Adrenal cortex; AME: Adrenal medulla; LIV: Liver, THY: Thyroid gland; DIA: Diaphragm; BIC: *Biceps femoris* muscle; AFT: Abdominal fat tissue; STO: Stomach; BLO: Blood; TES: Testes.

We have chosen the pig, not only because of its world-wide relevance in food production, but also because it is considered as one of the most important biomedical animal models [13]. Notably, the latest instalment of the EBI Gene Expression Atlas ([14]; http://www.ebi.ac.uk/gxa) with over 19 species, does not contain the pig.

Further, we develop a new methodology for the identification of tissue-specific genes. This methodology analyzes the tissue of the maximum expression of each gene and maintains the distribution of maximum expressed genes observed transcriptome-wide for each particular tissue. Additionally, we present the application of the PCIT algorithm [15] to construct a tissue specific regulatory network. Finally, we describe a novel use of the regulatory impact factor (RIF) metrics [16], [17] as a promising methodology for the search of TSTF in the whole transcriptome of an organism.

## Results and Discussion

### Quality Assessment of the Meta-Analysis Approach

The mixed-model used in the normalization accounted for 96.48% (goodness of fit, R^2^) of the total variation observed in the gene expression data. Ranked from more to less relevance, the main effect of gene accounted for 59.45%, followed by the interactions of gene by tissue (23.82%), gene by experiment (9.70%) and gene by array chip (3.51%).

**Figure 1:**
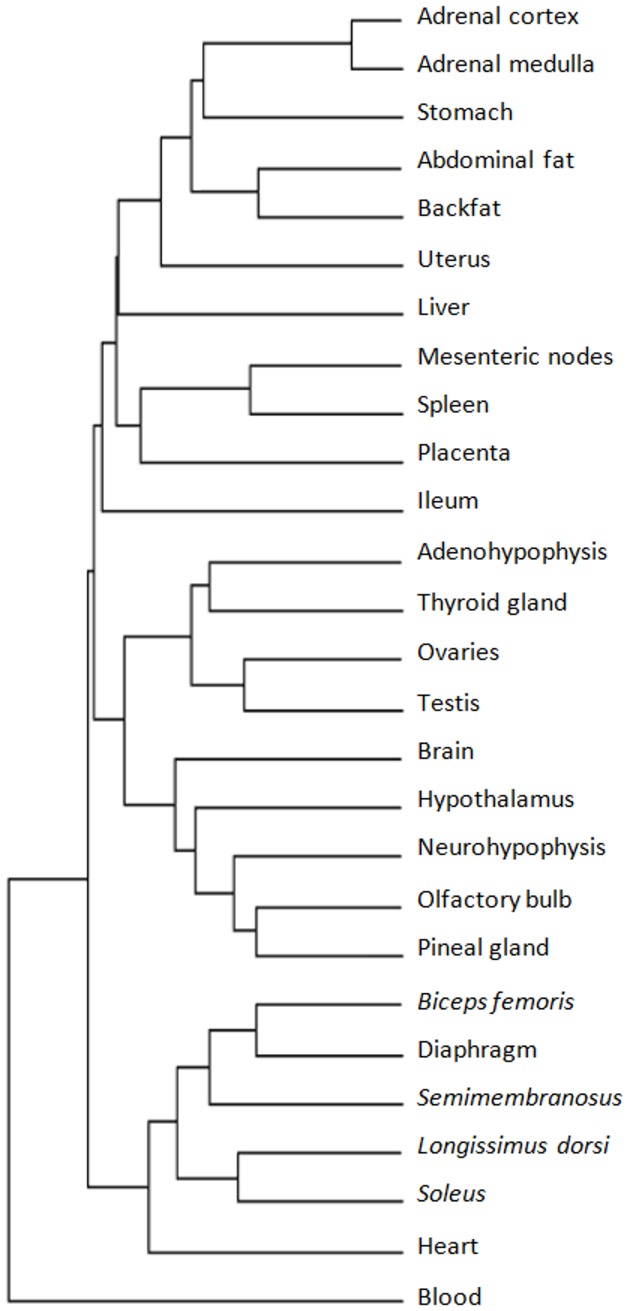
Clustering of tissues. Hierarchical cluster analysis of the 27 tissues based on the expression of 12,320 porcine genes.

The normalized mean expression of 12,320 genes across 134 experimental conditions was subjected to hierarchical cluster analysis using the PermutMatrix software [18]. Multiple experimental conditions of the same tissue clustered together, confirming the validity of operating at the level of tissue after averaging across the various conditions. Table S1 provides the compiled dataset with the normalised expression of 12,320 genes across the 27 tissues. In itself, this file represents the most comprehensive atlas of the porcine transcriptome published to date. Its content was also used as the input for the PermutMatrix software to generate the hierarchical cluster analysis of tissues presented in Figure 1. The tree resulting from the hierarchical cluster analysis of 12,320 genes across the 134 conditions is given in Figure S1. The fact that tissues clustered in an anatomical and functionally sensible manner (such as the clustering of the various skeletal muscles in one branch of the hierarchical tree and tissues from the central nervous system in another branch) was attributed to the optimality of the normalization process used in the meta-analysis and anticipates the confidence in the results that emerged in the subsequent analyses.

### Tissue-Specific Genes

Different methodologies for the identification of tissue-specific (TS) genes have been proposed. By and large, existing methods are direct functions of the ratio between the gene expression in one tissue to the sum total expression level across tissues. Our approach to identify TS genes combines the ratio of expressions with the distribution of the tissue location where the maximum expression of genes is observed (see Methods for details). A total of 1,234 (or 10%) of the genes were identified as TS.

**Figure 2:**
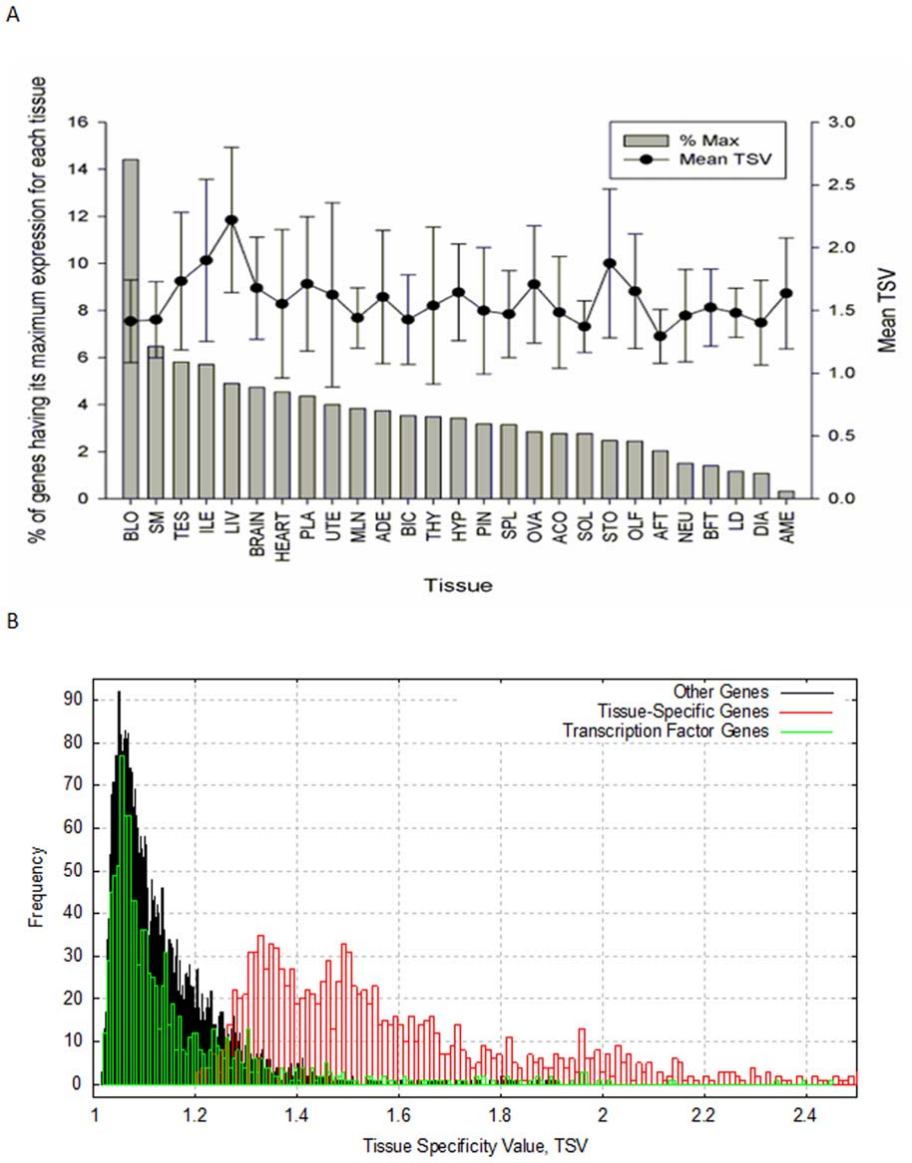
Tissue specificity value (TSV). (**A**) Distribution of the percentage of genes having its maximum expression in each tissue (left y axis) and the mean TSV of all the selected genes per tissue (right y axis). Standard errors are indicated as bars above and below the mean TSV. (**B**) Empirical density distribution of the TSV for tissue-specific genes (red bars), transcription factor genes (green bars) and remaining genes (black bars).

Figure 2A shows the distribution of the percentage of genes having its maximum expression in each tissue. By virtue of the methodology used to identify TS genes, this distribution was maintained when only the 1,232 TS genes were considered (see Materials and Methods for details in the identification of TS genes). There are noticeable differences in the proportion of genes having their maximum expression in the various tissues. On the one extreme, blood has by far the highest percentage of TS genes (14.4%) and this was attributed to blood representing a highly heterogeneous tissue with the haematopoietic cascade reported to result in the differentiation of very specific cell types [19], [20]. On the other extreme, adrenal medulla is the tissue with the lowest percentage of TS genes (0.3%), followed by two muscle tissues, *Longissimus dorsi* (1.1%) and diaphragm (1.1%). It should be noted that having multiple representatives of related tissues (eg. skeletal muscle and the central nervous system (CNS), each represented by six tissues) could affect the distribution of the tissue location of the TS genes. To overcome this potential artefact, Figure 2A also shows the mean of the tissue specificity value (TSV) of the TS genes in each tissue. Although with some oscillations, it is worth noting that this value remains similar across all tissues (overlaid trend in Figure 2A), and ranges from 1.3 (for liver) to 2.2 (for abdominal fat). Importantly, the distribution of the TSV for TS genes was found to be quite different from that of the TF genes or the remaining genes (Figure 2B).While low TSV were observed for the entire set of 12,320 genes as well as for the 1,072 TF genes only, higher and more spread TSV were observed for the set of 1,230 TS genes. In this respect, 90% of all genes had a TSV ranging from 1.036 to 1.532. Similarly, 90% of all TF had a TSV ranging from 1.043 to 1.542. However, the TSV observed for 90% of TS genes ranged from 1.280 to 2.230.

A gene ontology (GO) enrichment analysis of the 1,232 TS genes (target list) against all the 12,320 genes (background list) revealed *Multicellular Organismal Process* (GO:0032501) as the most enriched biological process (*P*-value = 8.25E-17, FDR *q*-value = 8.45E-13). Moreover, the second and the third most enriched biological processes were *System Process* (GO:0003008; *P*-value = 9.11E-17, FDR *q*-value = 4.67E-13) and *Developmental Process* (GO:0032502; *P*-value = 2.52E-16, FDR *q*-value = 8.60E-13), respectively. By definition, these enriched GO terms, are related to processes whose specific outcome is the progression of cell, tissues or organs (*Multicellular Organismal Process* and *Developmental Process*) or to processes carried out by organs or tissues in multicellular organisms (*System Process*). Given that multicellular organisms are organised into tissues, this result could be a reflection of the optimality of the numerical strategy used to identify TS genes. Also, there were four muscle related GO terms in the top ten enriched processes: *Muscle Filament Sliding* (GO:0030049; *P*-value = 2.17E-15, FDR *q*-value = 4.45E-12), *Actin-Myosin Filament Sliding* (GO:0033275; I-value = 2.17E15, FDR *q*-value = 3.71E-12), *Muscle System Process* (GO:0003012; *P*-value = 6.31E-14, FDR *q*-value = 8.08E-11) and *Muscle Contraction* (GO:0006936; *P*-value = 9.86E-14, FDR *q*-value = 1.12E-10). This could be reflecting the high proportions of skeletal muscle tissue types in our data.

In the last decade, tissue specificity of gene expression has been linked to a number of important attributes including, but not limited to level of expression [21], ability to detect cis- and trans- expression quantitative trait loci [22], differential rates of polymorphism [23], imprinting [24] and evolution [3], [4], as well as disease-association [5], [6] and sex biased [25]. Genomic imprinting is a genetic phenomenon by which certain genes are expressed in a parent-of-origin-specific manner [26]. Table 2 shows an enrichment of transcription factors, imprinted genes and disease-associated genes among the TS genes indentified in our study. Given this prior knowledge, the results from Table 2 provide further evidence of the optimality of the analytical approach taken here to identify TS genes.

**Table 2:** Enrichment of tissue-specific genes for transcription factors (TF), imprinted genes (IMP) and disease-associated genes (DIS).

	All genes (N = 12,320)		Tissue-Specific (N = 1,232)		*P*-value
	N	%^A^	N	%^A^	
TF	1,072	8.70	112	9.09	3.67E-02
IMP	134	1.09	23	1.87	3.53E-03
DIS	8,807	71.48	969	78.65	3.74E-10

A These percentages do not sum to one because not all of the 12,320 genes (or the subset of 1,232 tissue-specific genes) belong to one of the tree categories under scrutiny: TF, IMP and DIS.

### Tissue-Specific Regulatory Network

**Figure 3:**
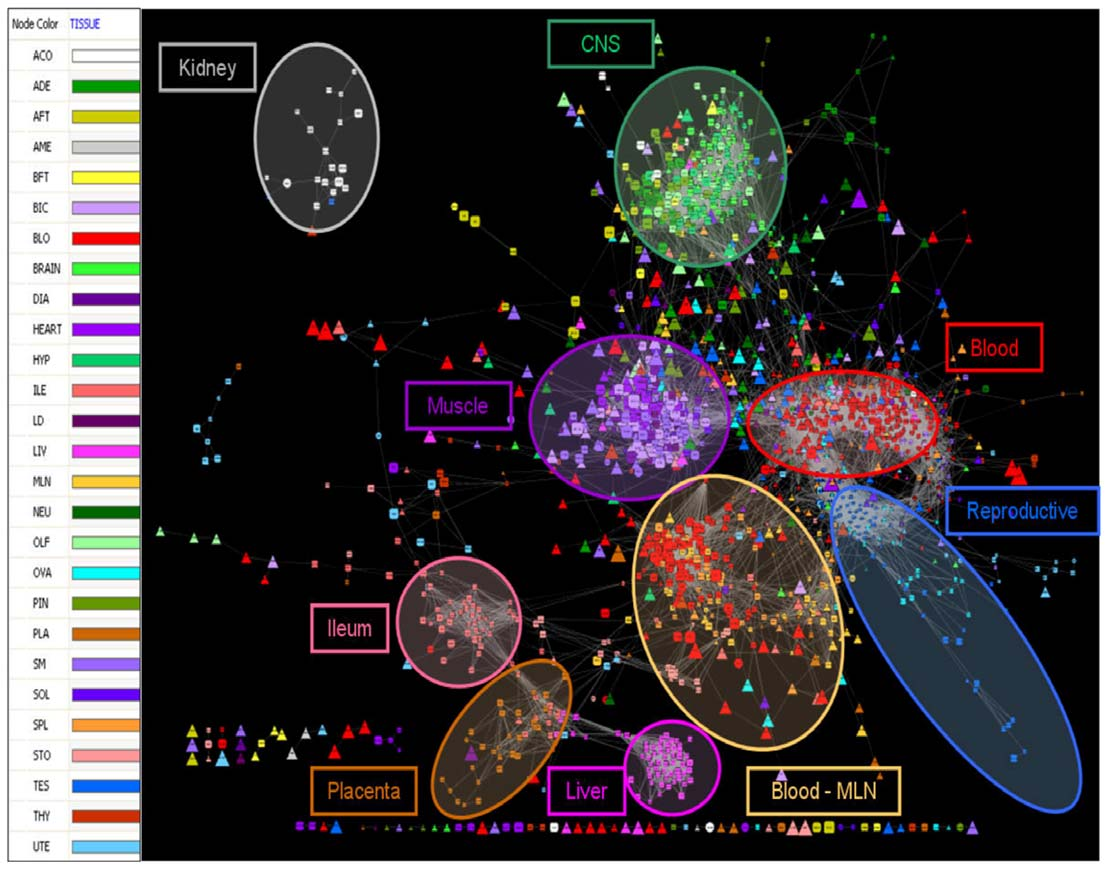
Tissue specific regulatory network of the porcine transcriptome. Legend of colours assigned to each of the 27 tissues in the network (left). The co-expression network (right). Node size was mapped to average transcript abundance, node colour was mapped to the tissue in which each particular gene is specific and node shape was mapped to the different gene types: TS (squares), TF (triangles) and TSTF (circles).

With the available 2,192 genes that included 1,120 TS, 960 TF and 112 TSTF genes, we reverse engineered a co-expression network. The overall network contained these 2,192 genes connected by 185,132 significant edges (or 7.7% of the possible 2,401,336 connections). The image of the network built from connections with significant correlation coefficients higher than 0.80 in absolute value is shown in Figure 3. This network comprises 1,572 nodes or genes and 20,084 edges or gene connections. Figure S2 contains the Cytoscape file created to access this network.

Within the whole network, several connected components could be distinguished: one big group composed of 1,461 connected nodes, two smaller ones formed by 21 and 8 nodes respectively (on the left of the image), and a large number of small groups containing 2 to 5 genes each (showed at the bottom of the image). When the tissue where a gene had its highest expression was mapped in the visualisation schema by assigning different colours to different tissues, it became immediately apparent that nodes clustered mainly by tissue. Most of the tissues, represented by different colours in Figure 3, appeared separated from each other as independent clusters, with the exception of the six muscle tissues that clustered together in one large module (purple colours, Figure 3). An identical observation can be made for the six tissues from the central nervous system (CNS) that clustered together in their own module (green colours, Figure 3).

The fact that each cluster represents a particular tissue was further confirmed by GO enrichment analyses. In doing so, biological processes enriched in the module assigned to ‘*muscle*’ included *Muscle Development* (*P*-value = 1.05E-26) and *Muscle Contraction* (*P*-value = 6.17E-24). Also, the module assigned to ‘*CNS*’ was enriched by *Nervous System Development* (*P*-value = 9.77E-12) and *Synaptic Transmission* (*P*-value = 1.53E-9). Similarly, the module formed by the mesenteric lymph nodes and the spleen was enriched for *Immune System* (*P*-value = 7.22E-17).

**Figure 4:**
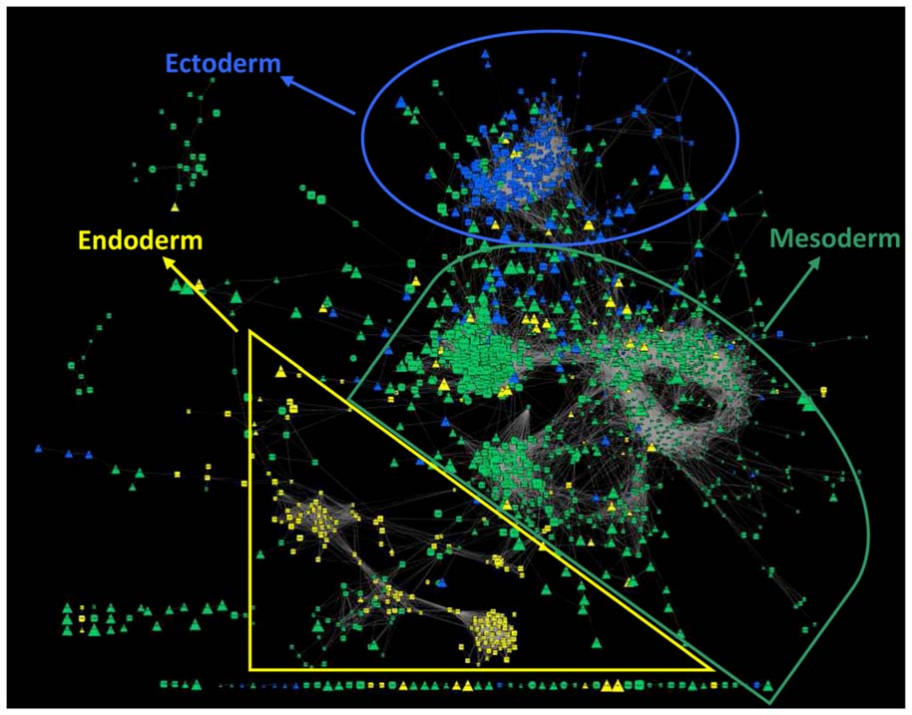
Embryonic origin of tissues. Tissue specific regulatory network of the porcine transcriptome, showing the embryonic origin of each tissue. In this instance, node colour was mapped to the embryonic origin of each tissue: blue for the ectoderm-derived tissues, green for the mesoderm ones and yellow for the tissues formed from the endoderm.

Moreover, if we colour each node by the embryonic origin of the tissue in which this gene is specific, it becomes apparent that tissues cluster according to their embryonic origin (Figure 4). For example, the tissues formed from the ectoderm (such as the CNS) were positioned at the top region of the network (in blue colour, Figure 4), the ones that originated from the mesoderm (case of the muscle, blood, adrenal cortex and medulla, gonads, spleen, mesenteric lymph nodes, uterus, placenta and fat) appeared in the centre of the network (in green colour, Figure 4), with the exception of the placenta that was located in the middle of the endoderm group, and the endoderm derived tissues (stomach, thyroid gland, ileum and liver) that were located in the bottom left part of the network (in yellow colour, Figure 4). Mesodermal tissues are overrepresented and more widespread in our network. Importantly, among the three germ layers, the mesoderm originated the last and its evolution is linked to the evolution of axis formation in metazoa and the appearance of eumetazoa. It is responsible for generating tissues specialized in protection, locomotion and sensing the environment that characterizes complex organisms [27].

The data and conclusions drawn from the network confirm its reliability and agreement with previous knowledge. The classification of tissues based on patterns of gene expression in the network largely reproduces classifications based on anatomical and biochemical properties [1]. Surprisingly, genes not only clustered by tissue in the network, but also, tissues clustered together according to their embryonic origin. This fact has already been noted in a mouse and human TF atlas [11] and can be attributed to these tissues being derived from transcriptional alteration of a common precursor and therefore expected to share large sections of expression patterns in common. The GO enrichment analysis provides further evidence about the quality of the inferred network and confirms that indeed it is a good representation of tissue specific regulation. Once we have confirmed the reliability of the results, we can be confident of their ability to allow for the extraction of downstream novel information about gene expression regulatory mechanisms.

### Tissue-Specific Transcription Factors (TSTF)

**Figure 5:**
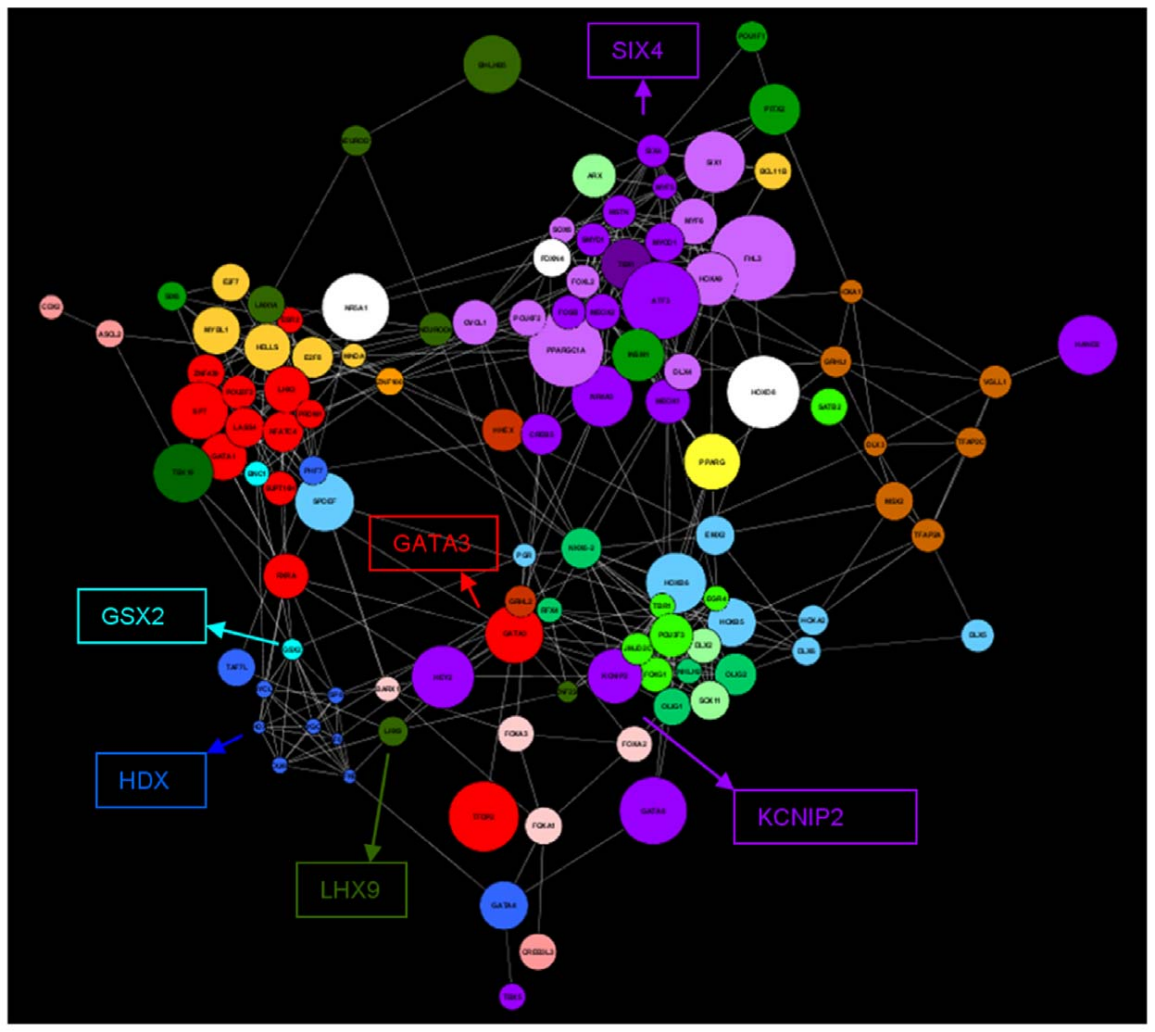
TSTF Network. The colour codes are as per Figure 3 and node size was mapped to average transcript abundance.

Based on their dual classification, the 112 TSTF genes were worthy of further analyses because these type of molecules provide excellent targets for targeted tissue therapies without broadly changing other tissues. However, the reader should bear in mind that tissue specificity is a continuous variable and that not all tissues at all time points were included in this analysis. In addition, most samples contain more than one different cell type, for example muscle contains contractile cells, adipocytes, nerve cells, blood cells and fibroblasts amongst other cell types. A search of the literature supported the tissue-specificity action for 71 of these 112 genes (Table S2). Importantly, they were distributed uniformly across the entire network, as opposed to showing a preference for a particular tissue (Figure 1 in Figure S2). When we focussed on the sub-network spanned by the TSTF genes, we identified 457 connections (Figure 5). Many interactions involving one TSTF gene were also confirmed in the literature and we highlight four. Firstly, *SIX4* appeared as a muscle specific TF in the network, directly connected to other fourteen TSTF. This TF is known to act as a regulator of *MYOD1*, the master regulator of the skeletal muscle gene expression program [28]. This role would explain the fact that *SIX4* is linked to nine muscle specific TF in the network. In addition, *SIX4* appeared also linked to four TSTF located in the CNS module of the network. Searching in the literature, we found that this could be attributed to *SIX4* playing a critical role controlling the formation of the olfactory embryonic epithelial layers and neuronal development [28]. Moreover, SIX4 carry out its action in the CNS acting synergistically with *SIX1* [29], to which *SIX4* was also connected in the network. Our second example is *KCNIP2*, a regulator of cardiac ionic currents [30] that appeared connected to several CNS genes in our network. Quite notably, *KCNIP2* plays a role in the regulation of neuronal excitability in response to intracellular ions [31]. For our third example, we emphasize *LHX9*, a pineal gland specific TF which in our network appeared connected to two testis-specific genes (*TAF7L* and *POU4F1*) and this TF has been shown to drive the axonal trajectory of some types of neurons [32] and also to play a role in gonadogenesis [33]. For our last example, we look at *GATA3*, a blood specific TF according to our tissue-specificity assignment and also related to brain and hypothalamus genes in our network. Significantly, *GATA3* has been shown to be required both in the regulation of hematopoietic stem cells [34] and in maintaining survival of the sympathetic neuron lineage [35].

All these findings support the idea that the TSTF network represents a reliable source for the generation of novel hypotheses regarding the key regulatory roles of these genes. One prominent example is the case of *GXS2*, highlighted in soft blue in Figure 5, with a total of 11 connections. This TF has not been previously associated with any reproduction or gonadal process. However, it was classified as ovary specific by our methodology, and connected in the network with the only other ovary specific gene, *BCN1*. Importantly, *BCN1* is found in abundance in the germ cells of ovaries [36]. Moreover, *GSX2* was connected to 8 more genes specific of reproductive tissues (testis and uterus). These observations support the novel hypothesis that, in addition to its known role in neuronal development in the forebrain [37], *GSX2* is a key regulator involved in gonad or reproductive processes. Similarly, *HDX*, highlighted in dark blue in Figure 5, has not been well described to date except its location on the X chromosome. In our network, this gene appeared as a testis specific TF connected with *POU4F1*, a known regulator involved in spermatogonia and expressed in distinct cell types in the testis [38], and joined to other 5 testis-specific TF. These findings suggest a potential role of *HDX* in testis development and/or function.

### Muscle and CNS Transcription Regulators

**Figure 6:**
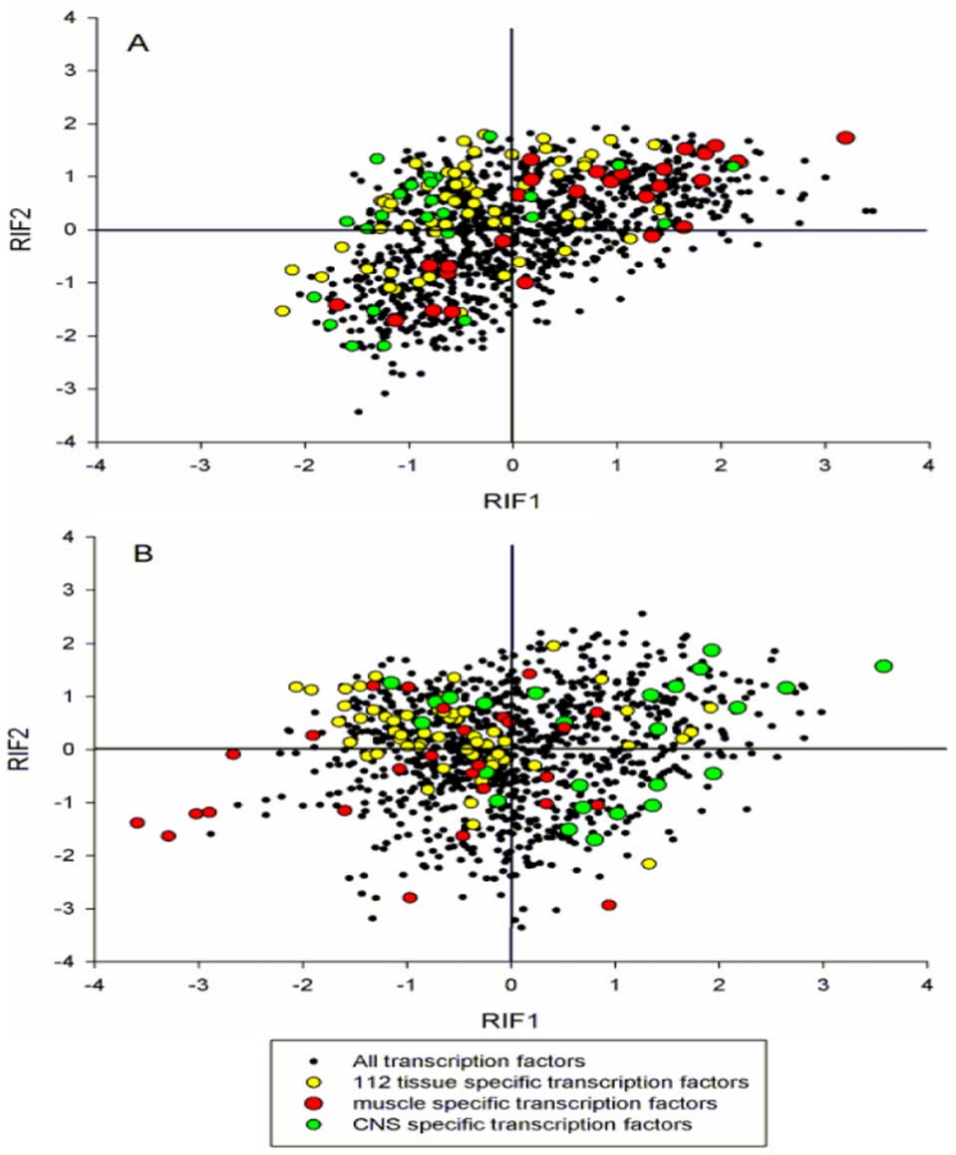
Regulatory impact factors (RIF). Scatter plot of the relationship between RIF1 and RIF2 in the two contrasts explored: (**A**) Muscle *vs.* Other Tissues; and (**B**) CNS *vs.* Other Tissues. Notice how in each contrast, the transcription factors of biological relevance are concentrated on the right half and upper-right quarter of the scatter.

In order to gain further insights into the identity of key regulators responsible for muscle and CNS differentiation and development, we undertook a series of regulatory impact factor (RIF) analyses. The aim of these analyses was to highlight those TF which, while might not be themselves differentially expressed or abundant, they still show differential connectivity, as measured by a big change in their co-expression correlation with the highly abundant highly differentially expressed genes. Figure 6 shows the relationship between RIF1 and RIF2 for all 1,072 TF in the two contrasts explored: ‘Muscle *vs.* Other Tissues’ (Figure 6A) and ‘CNS *vs.* Other Tissues’ (Figure 6B). The relevance of the RIF analyses became immediately clear when highlighting TF according to their tissue specificity. Muscle specific TF are highlighted in red, CNS specific TF in green and the rest of the 112 total TSTF are represented in yellow. In each contrast, the TF of biological relevance appeared preferentially located on the right half and upper-right quarter of the scatter.

Table 3 shows the results from the enrichment analysis of the TSTF of each particular tissue (muscle or CNS) when focused on the TF whose ranking is greater than 2 (based on |RIF1|+RIF2; see Methods) in each of the analyses. The ranked list of TF showed a significant enrichment of TSTF consistent with the contrast under scrutiny and more pronounced in muscle (*P*-value = 0.013) but also significant in the CNS analysis (*P*-value = 0.027). In the overall dataset, muscle-specific TF represented just a 2.5% of the total, however, when we focused on TF that showed values of |RIF1|+RIF2>2 in the ‘Muscle vs. Other Tissues’ comparison they represented a 5.3%. In the same way, CNS-specific TF represented only a 2.3% of the total TF, and a 4.7% of the TF with |RIF1|+RIF2>2 in the ‘CNS vs. Other Tissues” comparison. This enrichment of muscle and CNS TF in each particular analysis underscored the ability of the RIF algorithm to correctly identify the key regulators.

**Table 3:** Enrichment of tissue specificity in the regulatory impact factor (RIF) analysis.

	Overall		|RIF1|+RIF2>2			
			Muscle vs others		CNS vs Others	
	N	%	N	%	N	%
TF	960	89.5	151	88.3	151	88.3
TF CNS	25	2.3	3	1.7	8	4.7
TF muscle	27	2.5	9	5.3	5	3.0
TFTS	60	5.6	8	4.7	13	7.6
Total	1,072		171		177	

To further validate the performance of the RIF analyses, we searched for enriched GO terms in the ranked list of TF according to their combined RIF scores. In assessing the “Muscle *vs.* Other Tissues’ output we found that 8 of the top 10 most enriched biological processes were related to muscle function or development. Some of them include: “Cell migration involved in heart development” (*P-value* = 5.25E-5, FDR *q*-value = 1.21E-1) “ventricular cardiac muscle tissue development” (*P-value* = 1.21E-4, FDR *q*-value = 1.85E-1), “muscle tissue development” (*P-value* = 7.04E-4, FDR *q*-value = 4.64E-1) and “regulation of striated muscle cell apoptotic process (*P*-value = 8.5E-4, FDR *q*-value = 3.92E-1).

The fourteen TF contained in “muscle tissue development” were ranked as follows by RIF out of the 1,072 TF (rank shown in brackets): *TBX5* (1), *SIX1* (9), *MYF6* (24), *PPP1R13L* (29), *MYOD1* (30), *GATA4* (45), *HOCXD9* (72), *MYF5* (75), *FOXP2* (116), *ZNF238* (152), *EYA2* (179), *MYOG* (196), *TCF21* (199) and *OSR1* (200). Other TF correctly prioritised by RIF, but that were overlooked by the GO enrichment analyses include *MED12* (4) [39], *MYOCD* (6) [40], *LMO4* (7) [41] and *PITX2* (20) [42]. The most outstanding case would be the *TBX5*, as it is one of the most extreme TF according to both RIF scores. In addition, *ERCC3* was the second gene according to RIF1 score, and it was neither assigned as a muscle-specific TF by our analyses, nor has it been previously associated to muscle function. In addition, we found that the co-expression correlation of *ERCC3* gene with *MYOCD* of −0.768 was found to be significant by the PCIT algorithm. Based on these results, we could strongly suggest a novel key role of *ERCC3* as a muscle regulator.

To gain a further insight as to the reasons why *ERCC3* scored so highly according to the RIF algorithm, we explored its relationship with the differentially expressed genes. Table 4 lists the identity of the 10 most differentially expressed genes in the ‘Muscle *vs.* Other Tissues’ contrast. The values for the differential co-expression of *ERCC3* and *MYOCD* with the 10 most differentially expressed genes are also given in Table 4 (differential co-expression measured by the difference in correlation co-expression in muscle as compared to other tissues). Extreme values of differential co-expression were observed between *ERCC3* and a number of highly differentially expressed genes including *MYOZ2*, *MYOT*, *MYL1* and *TNNT1*, with differences of equivalent magnitude to those found for *MYOCD*, a well-known master regulator of cardiac and smooth muscle [40].

**Table 4:** Normalized mean expression (NME, base-2 logarithm scale) and differential expression (DE) for the 10 most DE genes along with their co-expression correlation with *ERCC3* and *MYOCD* in the skeletal muscle and other tissues.

Gene	NME	DE	Corr. with *ERCC3* in		Corr. with *MYOCD* in	
			Muscle	Others	Muscle	Others
*MYL2*	6.277	4.311	−0.467	−0.596	0.449	0.591
*MYOZ2*	4.447	4.187	−0.579	0.402	0.638	−0.422
*MYOT*	4.891	4.131	0.462	0.017	−0.462	0.087
*TTN*	5.637	4.069	−0.118	0.163	0.237	−0.133
*MB*	5.086	4.061	−0.471	−0.374	0.585	0.129
*CKM*	7.126	3.994	−0.155	0.128	0.146	0.018
*MYL1*	5.396	3.989	0.823	0.162	−0.944	0.134
*MYH7*	7.060	3.985	−0.463	−0.409	0.445	0.099
*ACTA1*	7.103	3.914	−0.394	−0.611	0.555	0.651
*TNNT1*	5.930	3.856	0.599	0.164	−0.747	−0.328

With respect to the RIF analysis of CNS tissues *versus* others, five out of the top ten regulators according to RIF are in fact involved in CNS development or function. The top one, *INSM1* is a neuroendocrine differentiation regulator [43], *RBL1* and *TSC22D4* are required for normal cerebellar development and differentiation [44], [45], *HBP1* regulates transcription in developing myeloid cells [46] and *SIX6* acts at the hypothalamus to control reproduction and fertility [47].

We noted that the ability of the RIF analysis to identify key regulators is equally satisfactory when contemplating two very different scenarios, muscle and CNS, correctly identifying five of the top ten regulators in both cases. While the muscle cell types are all very similar between the different muscles analysed and they all have a similar function, the CNS comprises a much more complex group of tissues that includes many different cell types and functions. We could highlight, for example, the existing differences between the pineal gland, an endocrine gland responsible for secreting different hormones, and the brain, that acts as the main coordinator of the entire CNS. The performance of RIF in these two very different circumstances indicates its generality.

### Concluding remarks

In conclusion, we assembled, curated and normalised a comprehensive collection of Affymetrix-based gene expression experiments in the pig in an attempt to better understand the transcriptional control of tissue development. This provided transcriptome data for 27 different tissues. Analogous approaches have been undertaken before in other species and more tissues. However, our study differs from these previous studies in two critical regards.

Firstly, we apply a set of higher-order network analyses in addition to the more conventional abundance ratio-based methods for determining tissue-specificity and tissue regulation. Because we do more than present a comprehensive survey of transcript abundances across tissues, our approach is more than a ‘Gene Atlas.’ Secondly, by focussing on the pig, we provide a new resource for a previously unexplored and yet important biomedical model and commercially-important livestock species.

Our meta-analysis approach was conducted according to the preferred reporting items for systematic reviews and meta-Analysis (PRISMA; http://prisma-statement.org/). However, as it is the case with all meta-analysis approaches, our study suffers from the inability to control for experimental design effects that may contribute to bias. The data used in the present work comes from studies exploring different breeds of pigs at various developmental stages. However, we advocate that optimal normalization approaches, such as those based on mixed-model equations, allow for the integration of seemingly disparate datasets such that the results are richer in information than any of the studies taken independently.

Researchers using gene expression technologies in the quest for systems-level explanations of biological phenomena are encouraged to explore holistic measures of differential connectivity in addition to differential expression [48]. Inspired by these holistic measures, we explored a combination of strategies that allowed us to identify not only tissue-specific genes but also their transcriptional regulators. Firstly, we developed and adapted an abundance ratio metric to assess tissue-specificity. Genes highlighted by this measure are abundantly expressed in that tissue relative to others, and the approach does not skew towards any particular tissue. Then, we used both co-expression (PCIT) and differential co-expression (RIF) approaches to prioritise regulatory molecules predicted to drive each tissue phenotype (i.e., its mature physical appearance), built on the numerical foundation provided by the initial tissue-specific metric.

Through significant co-expression to tissue-specific genes, the co-expression based approaches identified important tissue regulators that may themselves be only poorly or moderately expressed in that particular tissue. On the other hand, the RIF approach identifies regulators whose behaviour (connectivity) changes between two tissues, even though they may not be strongly co-expressed in either tissue or abundantly expressed in those tissues. The PCIT co-expression network and RIF analysis exploit the same numerical signals in different ways, and therefore complement each other. We advocate the use of the combination of approaches in order to gain as much regulatory information as possible from transcriptome data.

## Materials and Methods

### Description of Datasets

All the datasets used in this study are publicly available microarray gene expression experiments that have been deposited on the Gene Expression Omnibus (GEO) database ([49]; http://www.ncbi.nlm.nih.gov/geo/). We have selected only those using pig tissues, that used the Affymetrix platform and that were amenable to our purpose including those surveying anatomically defined tissues. We tried to capture as many tissues as possible and without any given tissue being over-represented.

Table 1 shows the GEO accession number and a brief description of the data sets. In total, they comprise 480 Porcine Affymetrix microarray experiments from 20 independent studies. Combined, they surveyed 134 experimental conditions across 27 tissues. These 27 tissues included six muscles tissues (*Semi-membranosus* (SM), *Longissimus dorsi* (LD), heart (HEART), diaphragm (DIA), *Biceps femoris* (BIC) and *Soleus* (SOL)), two fat tissues (abdominal fat tissue (AFT) and back fat tissue (BFT)), three reproductive tissues (ovaries (OVA), uterus (UTE) and testis (TES)), two kidney regions (adrenal medulla (AME) and adrenal cortex (ACO)), two hypophysis regions (adenohypohypisis (ADE) and neurohypophysis (NEU)), two glands (thyroid gland (THY) and pineal gland (PIN)), brain (BRAIN), ileum (ILE), placenta (PLA), spleen (SPL), mesenteric lymph nodes (MLN), olfactory bulb (OLF), hypothalamus (HYP), stomach (STO), liver (LIV) and blood (BLO).

### Data Processing, Quality Edits and Annotation

We obtained the MAS5 intensity signals (based-2 log expression) from all 480 microarray experiments (Table 1). The compiled data set includes many different conditions, pig breeds, ages and treatments. Therefore, a further location and scale normalization approach that took into account these features in a hierarchical fashion was deemed necessary [50]. To this effect, for each probe set (n = 24,124) the average intensity signal across biological replicates was computed resulting in 134 experimental conditions. This was followed by the computation of the average signal across the different conditions per tissue, ending with one expression value of each probe for each of the 27 tissues. The file with the normalised expression values across the 134 experimental conditions, and the file with the normalised expression values across the 27 tissues were processed by the PermutMatrix software to examine the hierarchical cluster analysis results. In those analyses we used default settings including the McQuitty's linkage method and the Euclidean distance calculation method.

Next, in order to adjust for possible heterogeneity of variance in expression signals due to tissue, we performed the Z-score normalization by tissue (ie. subtracting the tissue-specific average signal and dividing by the standard deviation of all signals in that tissue). Finally, to each z-score normalized signal we added the main effect of each probe set from its average signal across all tissues.

The original annotation of the Affymetrix Porcine chip dates from 2006 [51]. However, for the present work we used a more recent annotation (dated 2010) from the same authors and available at http://www4.ncsu.edu/~stsai2/annotation/2010-01-19_Affymetrix_Porcine_Annotation_tab_delimited.txt.

For the selection of probes, the following filtering process was applied: Firstly, we selected only those probes that were annotated to known genes. Secondly, for those genes represented by several probes sets, the probe set with the highest expression value, average across all tissues, was used as the representative of that gene, as this is supposed to be the most accurate one (i.e., high expression values tend to correspond to best quality signals). It is possible that different probe sets of the same gene represent different transcripts of that gene, but we have chosen this ‘one probe set – one gene’ filtering to simplify the analyses. This filtering process resulted with the z-cored normalized expression of a total of 12,320 genes across the 27 tissues (Table S1).

### Further Normalization via Mixed-Model Equations

Following previously described approaches for the normalization of gene expression data with a view to co-expression analyses [50], the following linear mixed-model was fitted to the data:
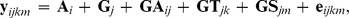
where 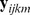 is the vector of MAS5 z-normalized gene expression signal for the *i*-th array chip hybridization, from the *j*-th gene in the *k*-th tissue and *m*-th experimental study; 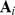 is the fixed effect of the *i*-th array chip hybridization (*i* = 1 to 480) and the fitting of which aims at normalizing the data by accounting for systematic non-genetic effects; 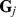 is the random component associated with the main effect of the *j*-th gene (*j* = 1 to 12,320); 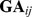 is the random component associated with the interaction between the *j*-th gene and the *i*-th array and it captures differences from overall averages that are attributable to specific gene by array combinations; 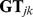 is the random component associated with the interaction between the *j*-th gene and the *k*-th tissue (*k* = 1, to 27) and it captures differences from overall averages that are attributable to specific gene by tissue combinations; 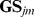 is the random component associated with the interaction between the *j*-th gene and the *m*-th experimental study (*m* = 1 to 20) and it captures differences from overall averages that are attributable to specific gene by study combinations; and 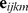 is the random residual error associated with 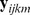.

Using standard statistical assumptions in mixed-model theory, the effects of 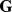, 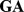, 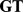, 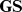 and 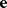 were assumed to be independent realizations from a normal distribution with zero mean and between-gene, between-gene within-array, between-gene within-tissue, between-gene within-study, and within-gene components of variance, respectively. Restricted maximum likelihood of variance components and solutions to model effects were obtained using the analytical gradients option of VCE6 software (ftp://ftp.tzv.fal.de/pub/vce6/).

### Transcription Factors (TF), Imprinted and Disease-Associated Genes

Next, among the genes included in our analyses, we were interested in identifying those being TF, and/or imprinted and/or disease associated. We resorted to the census of human TF [52] to identify 1,072 TF included in our dataset. In order to identify imprinting genes included in our dataset we mined the data from the following three publicly-available gene imprinting databases: MouseBook [53] (http://www.mousebook.org/catalog.php?catalog=imprinting), Catalogue of Parent of Origin Effects [54], [55] (http://igc.otago.ac.nz/home.html) and Geneimprint (http://www.geneimprint.com/site/genes-by-species). Similarly, disease-associated genes were identified as those annotated in the online Mendelian Inheritance in Man (OMIM) database (http://www.ncbi.nlm.nih.gov/Omim; [56]).

### Identification of Tissue-Specific Genes

Different methodologies for the identification of tissue specific genes (TS) have been proposed. Most of these methods use a direct function of the ratio between the gene expression in a particular tissue to the sum total expression level across tissues [1], [5]. However other tissue specificity measures that involved more complex components as the relative entropy have been presented [11]. Here, we describe a multi-tiered approach to identify TS genes. The algorithm proceeded as follows:

- Step 1: For each gene, note the tissue of its maximum expression. In formal terms, let *m_i_* be the identity of the tissue where the *i*-th gene shows its maximum expression, where *i* = 1, 2, …, *N* = 12,320.
- Step 2: For each tissue in *j*, compute *p_j_* = the percentage of genes having its maximum expression in it. In formal terms, define
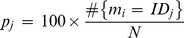
where *ID_j_* indicates the identity of the *j*-th tissue and *j* = 1, 2, …, 27.
- Step 3: Again for each gene, define and compute its tissue specificity value (TSV*_i_*) as the ratio between its maximum expression and its mean expression averaged across all 27 tissues. Accordingly:
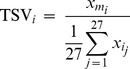
where 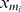 represents the expression of the *i*-th gene in tissue *m_i_*, and 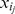 is the expression of the *i*-th gene in the *j*-th tissue.
- Step 4: Apply a nominal threshold of 10% by which 10% (or 1,232) of all genes in the dataset were deemed to be TS. Identify TS genes by maintaining the distribution of maximum expressions observed in the whole transcriptome. In formal terms, 10% of all *p_j_* were assigned as TS genes after selecting based on their TSV*_i_*.

For instance, if 6% of genes (or 740 out of 12,320) had their maximum expression in a given tissue, we selected the top 10% (or 74) of these 740 based on their TSV as TS genes. After applying this rationale to all tissues, we ended up with 10% of genes being tissue-specific and where the distribution of the location of their maximum expression was identical as that from the entire set of 12,320 genes.

### Network Inference and Visualization

The expression of genes that were annotated as TF and/or TS was used to reverse-engineer a gene co-expression network using the PCIT algorithm [15]. This algorithm combines the twin concepts of partial correlation coefficient with information theory to identify significant gene to gene co-expressions, defining edges in the re-construction of the network. It works by comparing the co-expression arrangements for triplets of genes, with all triplets being exhaustively explored, and providing the correlation estimate for each pair of genes together with a flag as to whether or not the estimate has been found to be significantly different from zero. Significant correlations establish an edge in the reconstruction of the network.

Although PCIT is a soft-thresholding method, it is possible to construct networks with more or less depth using different cut offs of the absolute value of the correlation co-expression among those found to be significant. Here we present a network built with absolute co-expression correlations greater than 0.80 among those found significant by the PCIT algorithm. We have used Cytoscape version 2.6.1 [57] to visualize the network and identify modules of co-expressed genes. The organic clustering algorithm that groups together genes with common neighbours was used to visualise the topology of the network. An additional network containing only the TSTF genes was built using the orthogonal Cytoscape layout. Gene ontology (GO) enrichment analyses of modules of co-expressed genes were performed within Cytoscape using the BinGO plug-in [58].

### Identification of Key Regulators: Case Study with Skeletal Muscle and the CNS

We used the Regulatory Impact Factor (RIF) metrics [16], [17] to identify critical muscle and central nervous system (CNS) TF from our gene expression data. The RIF metrics identify the regulators with the highest evidence of contributing to differential expression in two biological conditions. It yields two alternate measures of TF importance, RIF1 and RIF2. The RIF1 score highlights the transcriptional regulators most differentially connected to the most abundant differentially expressed genes, while the RIF2 measure highlights those TF with the most altered ability to act as predictors of the abundance of differentially expressed genes.

While the original implementation of the RIF metrics involved the comparison of the TF with the differentially expressed genes, the exact same algebra can be adapted to the comparison of the TF with the TS genes (or any other group of genes for that matter) as long as an experimental contrast is defined (eg. Condition A *vs.* Condition B). In this respect, for the RIF analyses, we explored two contrasts: In the first one, we compared the six muscle tissues (SOL, BIC, LD, HEART, SM and DIA) against the 21 other tissues. In the second contrast, we compared the six CNS tissues (ADE, NEU, BRAIN, OLF, HYP and PIN) against the others 21 tissues. Accordingly, the RIF metrics for the *i*-th TF (*i* = 1, 2, …, 1072) were computed using the following formulae:
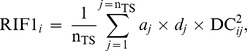
and
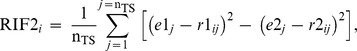
where n_TS_ is the number of TS genes (ie. n_TS_ = 1,232); *a_j_* is the abundance of the *j*-th TS gene as given by its normalised mean expression averaged across all tissues; *d_j_* is the differential expression of the *j*-th TS genes and computed from the difference between the expression of the *j*-th gene in the muscle minus its expression in the other tissues (for the first contrast), or from the difference between the expression of the *j*-th gene in the CNS minus its expression in the other tissues (for the second contrast); DC_ij_ is the differential co-expression between the *i*-th TF and the *j*-th TS gene, and computed from the difference between *r*1*_ij_*, the correlation co-expression between the *i*-th TF and the *j*-th TS gene in the muscle tissues (or in the CNS tissues for the second contrast), and *r*2*_ij_*, the correlation co-expression between the *i*-th TF and the *j*-th TS gene in the remaining tissues; and *e*1*_j_* and *e*2*_j_* represent the normalised mean expression of the *j*-th TS gene averaged across all muscle tissues (or across all CNS tissues for the second contrast) and across all the remaining tissues, respectively.

Importantly, RIF1 depends on the direction of (or which condition is used first in) the contrast, “A *versus* B” or “B *versus* A”. Instead, the sign of RIF2 is not affected by this contrast directionality, but by the change in the ability of the TF to predict the abundance of DE in the two conditions, regardless of which condition is considered first in the contrast. For this reason, we ranked TF based on their |RIF1|+RIF2 score. Finally, the ranked list of TF was processed through the GOrilla tool [59] to search for enriched GO terms. From this tool, we report the enrichment *P*-value computed from the hypergeometric test and the false discovery rate (FDR) *q*-value which corresponds to the p-value corrected for multiple testing using the Benjamini and Hochberg method [60].

## Supporting Information

Figure S1 **Hierarchical cluster analysis of the 143 experimental conditions based on the expression of the 12,320 porcine genes.**
(TIF)

Figure S2 **Cytoscape formatted file to allow the visualization and recreation of the networks presented in this study.**
(GZ) Click here for additional data file.

Table S1 **Comma delimited file with the normalized mean expression of 12,320 genes across the 27 tissues.**
(CSV)

Table S2 **Word document file listing the 112 tissue specific transcription factor genes, their expression and their location in the network.**
(DOC)
