# Supplementary figures and images for: Porcine Tissue-Specific Regulatory Networks Derived from Meta-Analysis of the Transcriptome
Source: PLoS One. 2012 Sep 26;7(9):e46159. doi: 10.1371/journal.pone.0046159 (PMC3458843; doi:10.1371/journal.pone.0046159)

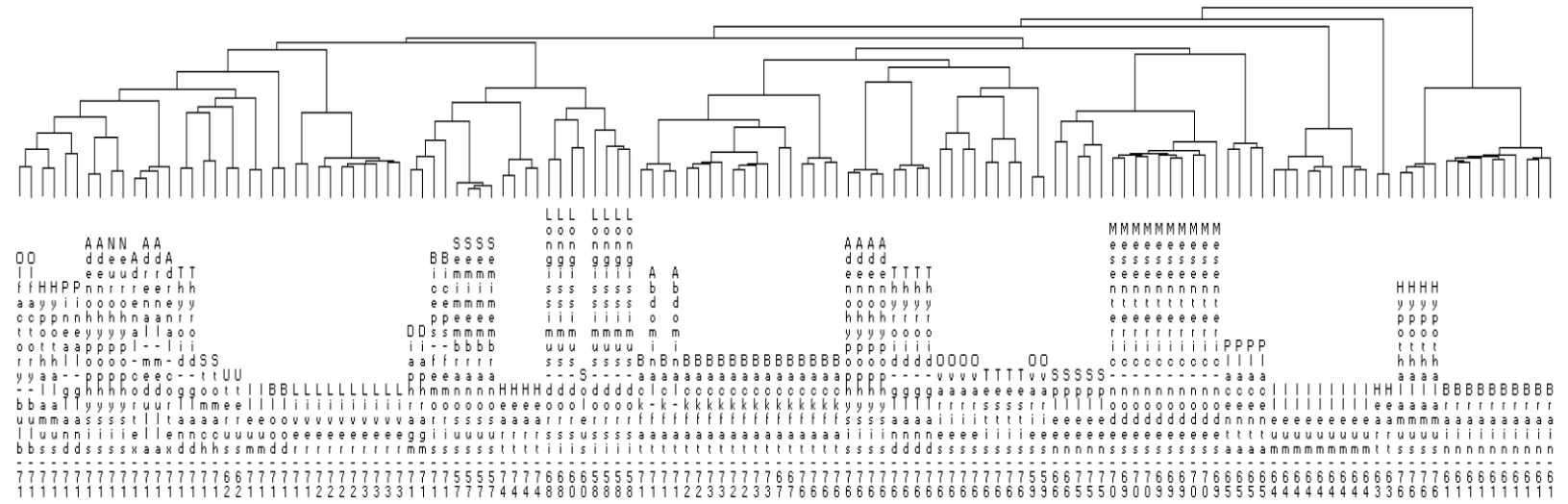

Supplement: Figure S1 — Hierarchical cluster analysis of the 143 experimental conditions based on the expression of the 12,320 porcine genes. (TIF) [file pone.0046159.s001.tif]
