# Supplementary material for: Porcine Tissue-Specific Regulatory Networks Derived from Meta-Analysis of the Transcriptome
Source: PLoS One. 2012 Sep 26;7(9):e46159. doi: 10.1371/journal.pone.0046159 (PMC3458843; doi:10.1371/journal.pone.0046159)

**Porcine Tissue-Specific Regulatory Networks Derived from Meta-Analysis of the Transcriptome**

D Pérez-Montarelo, NJ Hudson, AI Fernández, Y Ramayo-Caldas, BP Dalrymple, and A Reverter

**Additional File 3 – Tissue-Specific Transcription Factors (TSTF)**

**Table 1.** List of the predicted 112 tissue-specific transcription factors (TSTF) with their maximum and average expression, tissue in which they are specific, tissue specificity value (TSV) and the reference that confirms its tissue location.

| **Gene** | **Max** | **Average** | **Tissue** | **TSV** | **Reference^A^** |
| --- | --- | --- | --- | --- | --- |
| FOXN4 | 4.00 | 3.15 | ACO | 1.27 |  |
| HOXD8 | 8.29 | 6.49 | ACO | 1.28 | Doi-Poi et al., 2007 |
| NR5A1 | 7.91 | 5.97 | ACO | 1.32 | Ferraz-de-Souza et al., 2011 |
| INSM1 | 7.31 | 4.52 | ADE | 1.61 | Lan and Breslin, 2009 |
| PITX2 | 7.07 | 4.40 | ADE | 1.61 | Quentien et al., 2006 |
| POU1F1 | 4.59 | 2.48 | ADE | 1.85 | Lee et al., 2011 |
| SIX6 | 4.50 | 2.29 | ADE | 1.97 | Li et al., 2002 |
| PPARG | 7.12 | 4.90 | BFT | 1.45 | Roberts et al., 2011 |
| ESR2 | 2.53 | 1.94 | BLO | 1.30 | Scariano et al., 2008 |
| GATA1 | 5.25 | 4.02 | BLO | 1.31 | Zhang et al., 2000 |
| GATA3 | 6.77 | 5.07 | BLO | 1.33 | Buza-Vidas et al., 2011 |
| LASS4 | 4.29 | 3.26 | BLO | 1.32 | Hicks et al., 2009 |
| LHX3 | 5.16 | 3.92 | BLO | 1.32 |  |
| NFATC4 | 4.49 | 3.35 | BLO | 1.34 | Graef et al., 2001 |
| POU2F2 | 3.60 | 2.76 | BLO | 1.31 | Herbeck et al., 2011 |
| PRDM1 | 2.99 | 2.22 | BLO | 1.35 | Wilkinson et al., 2011 |
| RXRA | 4.93 | 3.83 | BLO | 1.29 | Stephensen et al., 2007 |
| SP7 | 6.62 | 4.97 | BLO | 1.33 | Valenti et al., 2008 |
| SUPT16H | 3.54 | 2.72 | BLO | 1.30 |  |
| TFDP2 | 9.33 | 6.24 | BLO | 1.49 |  |
| ZNF430 | 3.96 | 3.05 | BLO | 1.30 |  |
| EGR4 | 3.81 | 1.89 | BRAIN | 2.01 | Ludwin et al., 2011 |
| FOXG1 | 5.62 | 2.82 | BRAIN | 1.99 | Dastidar et al., 2011 |
| JMJD2C | 4.42 | 2.52 | BRAIN | 1.75 |  |
| POU3F3 | 6.25 | 3.63 | BRAIN | 1.72 | Sugitani et al., 2002 |
| SATB2 | 4.65 | 3.02 | BRAIN | 1.54 | Gyorgy et al., 2008 |
| TBR1 | 4.55 | 1.86 | BRAIN | 2.45 | Mckenna et al., 2011 |
| TBX1 | 5.92 | 4.32 | DIA | 1.37 |  |
| GATA6 | 8.08 | 5.99 | HEART | 1.35 | Maitra et al., 2010 |
| HAND2 | 7.29 | 5.22 | HEART | 1.40 | Shen et al., 2010 |
| HEY2 | 7.21 | 5.52 | HEART | 1.31 | Koibuchi et al., 2007 |
| KCNIP2 | 6.88 | 4.72 | HEART | 1.45 | Thomsen et al., 2009 |
| NR4A3 | 6.97 | 5.35 | HEART | 1.30 |  |
| TBX5 | 3.95 | 2.02 | HEART | 1.96 | Wang et al., 2011 |
| NHLH2 | 3.46 | 1.90 | HYP | 1.81 | Vella et al., 2007 |
| NKX6-2 | 5.09 | 3.38 | HYP | 1.51 |  |
| OLIG1 | 6.03 | 3.08 | HYP | 1.96 | Virard et al., 2006 |
| OLIG2 | 5.84 | 3.53 | HYP | 1.66 | Sun et al., 2011 |
| RFX4 | 3.64 | 1.92 | HYP | 1.90 | Zhang et al., 2007 |
| ASCL2 | 3.43 | 2.18 | ILE | 1.57 | Van der Flier et al., 2009 |
| CDX2 | 3.88 | 1.81 | ILE | 2.14 | Coskun et al., 2011 |
| CREB3L3 | 5.74 | 3.05 | ILE | 1.88 | Li et al., 2009 |
| MEOX2 | 3.99 | 2.89 | LD | 1.38 | Otto et al., 2010 |
| MSTN | 4.18 | 2.67 | LD | 1.57 | Hennebry et al., 2009 |
| SIX4 | 3.87 | 2.64 | LD | 1.46 | Aziz et al., 2010 |
| BCL11B | 5.06 | 3.06 | MLN | 1.65 | Durum 2003 |
| E2F7 | 4.33 | 3.10 | MLN | 1.39 |  |
| E2F8 | 4.84 | 3.43 | MLN | 1.41 |  |
| HELLS | 5.45 | 3.97 | MLN | 1.37 | Geiman and Muegge, 2000 |
| MNDA | 2.89 | 1.79 | MLN | 1.61 |  |
| MYBL1 | 5.49 | 4.06 | MLN | 1.35 | Golay et al., 1997 |
| TBX19 | 6.95 | 5.22 | NEU | 1.33 | Davis et al., 2010 |
| ARX | 5.47 | 3.68 | OLF | 1.49 | Yoshihara et al., 2005 |
| DLX2 | 4.95 | 2.83 | OLF | 1.75 | Brill et al., 2008 |
| SOX11 | 5.04 | 3.26 | OLF | 1.54 |  |
| BNC1 | 2.81 | 1.85 | OVA | 1.52 | Luchi et al., 1999 |
| GSX2 | 2.50 | 1.48 | OVA | 1.70 |  |
| BHLHB5 | 6.80 | 5.08 | PIN | 1.34 | Brunelli et al., 2003 |
| LHX9 | 3.41 | 2.34 | PIN | 1.46 | Avraham et al., 2009 |
| LMX1A | 4.51 | 3.10 | PIN | 1.45 |  |
| NEUROD1 | 4.74 | 2.41 | PIN | 1.97 | Munoz et al., 2007 |
| NEUROD4 | 5.20 | 2.72 | PIN | 1.91 |  |
| ZNF224 | 2.40 | 1.52 | PIN | 1.57 |  |
| DLX3 | 3.60 | 1.69 | PLA | 2.13 | Chui et al., 2010 |
| GRHL1 | 3.73 | 2.50 | PLA | 1.49 | Henderson et al., 2008 |
| HOXA13 | 4.09 | 1.75 | PLA | 2.34 | Shaut et al., 2008 |
| MSX2 | 5.47 | 3.16 | PLA | 1.73 | Quinn et al., 2008 |
| TFAP2A | 4.90 | 2.69 | PLA | 1.82 | Biadasiewicz et al., 2011 |
| TFAP2C | 3.80 | 2.39 | PLA | 1.59 | Kuckenberg et al., 2010 |
| VGLL1 | 4.85 | 2.59 | PLA | 1.87 |  |
| DLX4 | 4.03 | 3.04 | SM | 1.32 |  |
| FHL3 | 9.84 | 7.74 | SM | 1.27 | Cottle et al., 2007 |
| FOXL2 | 3.94 | 2.70 | SM | 1.46 |  |
| HOXA9 | 6.80 | 4.62 | SM | 1.47 |  |
| MYF6 | 6.39 | 3.93 | SM | 1.62 | Ropka-Molik et al., 2011 |
| OVOL1 | 4.91 | 3.84 | SM | 1.28 |  |
| POU6F2 | 4.08 | 3.03 | SM | 1.35 |  |
| PPARGC1A | 8.59 | 6.73 | SM | 1.27 | Lee et al., 2011 |
| SIX1 | 7.71 | 5.40 | SM | 1.43 | Gianakopoulos et al., 2011 |
| SOX6 | 2.73 | 2.01 | SM | 1.97 | An et al., 2011 |
| ATF3 | 9.21 | 6.94 | SOL | 1.33 |  |
| CREB5 | 4.33 | 3.43 | SOL | 1.26 |  |
| FOSB | 4.15 | 2.69 | SOL | 1.54 |  |
| MEOX1 | 4.93 | 3.77 | SOL | 1.31 |  |
| MYF5 | 2.97 | 1.81 | SOL | 1.64 | Francetic and Li, 2011 |
| MYOD1 | 5.00 | 3.21 | SOL | 1.56 | Aziz et al., 2010 |
| SMYD1 | 4.04 | 2.68 | SOL | 1.50 | Just et al., 2011 |
| ZNF100 | 3.60 | 2.14 | SPL | 1.68 |  |
| BARX1 | 3.23 | 1.88 | STO | 1.72 | Kim et al., 2011 |
| FOXA1 | 5.13 | 3.09 | STO | 1.66 | Ye et al., 2009 |
| FOXA2 | 5.66 | 2.92 | STO | 1.94 | Ye et al., 2009 |
| FOXA3 | 5.28 | 2.95 | STO | 1.79 |  |
| GATA4 | 6.11 | 4.12 | TES | 1.48 | Jing et al., 2009 |
| HDX | 1.38 | 0.72 | TES | 1.90 |  |
| MYCL1 | 1.63 | 1.11 | TES | 1.46 | Robertson et al., 1991 |
| NFKB1 | 1.35 | 0.77 | TES | 1.76 |  |
| PHF7 | 3.47 | 2.23 | TES | 1.55 | Xiao et al., 2002 |
| POU4F1 | 1.54 | 1.03 | TES | 1.49 | Budhram-Mahadeo et al., 2001 |
| PYGO1 | 1.42 | 0.93 | TES | 1.54 |  |
| SP8 | 1.89 | 1.03 | TES | 1.84 |  |
| TAF7L | 5.18 | 3.08 | TES | 1.68 | Akinloye et al., 2007 |
| ZNF627 | 1.33 | 0.55 | TES | 2.40 |  |
| GRHL2 | 4.56 | 2.52 | THY | 1.81 |  |
| HHEX | 4.92 | 3.60 | THY | 1.36 | Fagman and Nillson, 2011 |
| DLX5 | 5.14 | 2.62 | UTE | 1.96 |  |
| DLX6 | 4.41 | 2.25 | UTE | 1.96 |  |
| EMX2 | 5.13 | 3.26 | UTE | 1.52 | Taylor et al., 2005 |
| HOXA2 | 3.54 | 2.38 | UTE | 1.49 |  |
| HOXB5 | 6.07 | 4.16 | UTE | 1.46 |  |
| HOXB6 | 7.61 | 5.31 | UTE | 1.43 |  |
| PGR | 3.02 | 1.71 | UTE | 1.76 | Lee et al., 2006 |
| SPDEF | 7.11 | 5.17 | UTE | 1.38 |  |

**^A^Reference list:**

Akinloye,O et al. (2007) Mutation analysis of the X-chromosome linked testis-specific TAF7L gene in spermatogenic failure. Andrologia, 39, 190-195.

An,CL, Dong,Y and Hagiwara,N (2011) Genome-wide mapping of Sox6 binding sites in skeletal muscle reveals both direct and indirect regulation of muscle terminal differentiation by Sox6. BMC Dev. Biol., 11, 59.

Avraham,O et al. (2009) Transcriptional control of axonal guidance and sorting in dorsal interneurons by the Lim-HD proteins Lhx9 and Lhx1, Neural Development, 4.

Aziz A., Liu Q.-C. and Dilwort, F.J. (2010) Regulating a master regulator Establishing tissue-specific gene expression in skeletal muscle, Epigenetics, 5, 691-695.

Biadasiewicz,K et al. (20119 Transcription factor AP-2. promotes EGF-dependent invasion of human trophoblast. Endocrinol., 152, 1458-1469.

Brill,MS et al. (2008) A Dlx-2 and Pax6-dependent transcriptional code for periglomerular neuron specification in the adult olfactory bulb. J. Neurosci., 28, 6439-6452.

Brunelli,S., Innocenzi,A. And Cossu,G. (2003) Bhlhb5 is expressed in the CNS and sensory organs during mouse embryonic development. Gene Expr. Patterns, 3, 755-759.

Budhram-Mahadeo,V et al. (2001) The closely related POU family transcription factors Brn-3a and Brn-3b are expressed in distinct cell types in the testis. Intern. J.Biochem. Cell Biol. 33: 1027-1039.

Buza-Vidas N., et al. (2011) GATA3 is redundant for maintenance and self-renewal of hematopoietic stem cells, Blood, 118, 1291-1293.

Chui,A et al. (2010) Homeobox gene distal-less 3 is expressed in proliferating and differentiating cells of the human placenta. Placenta, 31, 691-697.

Coskun,M, Troelsen,JT and Nielsen,OH (2011) The role of CDX2 in intestinal homeostasis and inflammation. Biochim. Biophys. Acta, 1812, 283-289.

Cottle,DL et al. (2007) FHL3 binds MyoD and negatively regulates myotube formation. J. Cell Sci., 120, 1423-1435.

Dastidar,SG, Landrieu,PMZ and D.Mello,S (2011) FoxG1 promotes the survival of postmitotic neurons. J. Neurosci., 31, 402-413.

Davis,SW et al. (2010) Molecular mechanisms of pituitary organogenesis: in search of novel regulatory genes. Mol. Cell Endocrinol., 323, 4-19.

Di-Poi,N, ZÃ¡ny,J and Duboule,D (2007) Distinct roles and regulations for Hoxd genes in metanephric kidney development. PLoS Genet., 3, e232.

Durum,SK (2003) Bcl11: sibling rivalry in lymphoid development. Nat. Immunol., 4, 512-514.

Fagman,H and Nilsson,M (2011) Morphogenetics of early thyroid development. J. Mol. Endocrinol., 46, R33-42.

Ferraz-de-Souza,B et al. (2011) Sterol O-acyltransferase 1 (SOAT1, ACAT) is a novel target of steroidogenic factor-1 (SF-1, NR5A1, Ad4BP) in the human adrenal. J. Clin. Endocrinol. Metab., 94, E663-E668.

Francetic,T and Li,Q (2011) Skeletal myogenesis and Myf5 activation. Transcription, 2, 109-114.

Geiman,TM and Muegge,K (2000) Lsh and SNF2/helicase family member, is required for proliferation of mature T lymphocytes. Proc. Nat. Acad. Sci., 97, 4772-4777.

Gianakopoulos,PJ et al. (2011) MyoD directly up-regulates premyogenic mesoderm factors during induction of skeletal myogenesis in stem cells. J. Biol. Chem., 286, 2517-2525.

Golay,J et al. (1997) The A-myb transcription factor in neoplastic and normal B cells. Leuk. Lymphoma, 26, 271-279.

Graef,I.A., et al. (2001) Signals transduced by Ca(2+)/calcineurin and NFATc3/c4 pattern the developing vasculature. Cell, 105, 863-875.

Gyorgy,AB et al. (2008) SATB2 interacts with chromatin-remodeling molecules in differentiating cortical neurons. Eur. J. Neurosci., 27, 865-873.

Henderson,YC et al. (2008) LBP1b, LBP9 and LBP32/MGR detected in syncytuitrophoblasts from first-trimester human plancental tissue and their transcriptional regulation. DNA Cell Biol., 27, 71-79.

Hennebr,A et al. (2009) Myostatin regulates fiber-type composition of skeletal muscle by regulating MEF2 and MyoD gene expression. Am. J. Physiol., Cell Physiol., 296, C525-C534.

Herbeck,R et al. (2011) B-cell transcription factors Pax-5, Oct-2, BOB.1, Bcl-6 and MUM1 are useful markers for the diagnosis of nodular lymphocyte predominant Hodgkin lymphoma. Rom. J. Morphol Embryol., 52, 69-74.

Hicks,A.A. et al. (2009) Genetic determinants of circulating sphingolipid concentrations in European populations. PLoS Genet., 5, e1000672.

Jing,CX et al. (2009) The GATA family in reproduction. Zhonghua Nan Ke Xue, 15, 932-936.

Just,S et al. (2011) The myosin-interacting protein SMYD1 is essential for sarcomer organization. J. Cell Sci., 124, 3127-3136.

Kim,BM et al. (2011) Regulation of mouse stomach development and Barx1 expression by specific microRNAs. Development, 138, 1081-1086.

Koibuchi,N and Chin,MT (2007) CHF1/Hey2 plays a pivotal role in left ventricular maturation through suppression of ectopic atrial gene expression. Circ. Res., 100, 850-855.

Kuckenberg,P et al. (2010) The transcription factor TCFAP2C/AP-2gamma cooperates with CDX2 to maintain trophectoderm formation. Mol. Cell Biol., 30, 3310-3320.

Lan M.S. and Breslin, M.B. (2009) Structure, expression, and biological function of INSM1 transcription factor in neuroendocrine differentiation, FASEB Journal, 23, 2024-2033.

Lee,JS et al. (2011) Effects of polymorphisms in the 3.untranslated region of the porcine PPARGC1A gene on muscle fiber characteristics and meat quality traits. Mol. Biol. Rep., [Epub 2011 Jul 12].

Lee,K et al. (2006) Molecular mechanisms involved in progesterone receptor regulation of uterine function. J. Steroid. Biochem. Mol. Biol., 102, 41-50.

Lee,NC et al. (2011) Congenital hypopituitarismd due to POU1F1 gene mutation. J. Formos. Med. Assoc., 110, 58-61.

Li,X et al. (2002) Tissue-specific regulation of retinal and pituitary precursor cell proliferation. Science, 297, 1180-1183

Li,X et al. (2009) Dynamic patterning at the pylorus: formation of an epithelial intestine-stomach boundary in late fetal life. Dev. Dyn., 238, 3205-3217.

Luchi,S and Green,H (1999) Basonuclin, a zinc finger protein of keratinocytes and reproductive germ cells, binds to the rRNA gene promoter. Proc. Nat. Acad. Sci., 96, 9628-9632.

Ludwig,A et al. (2011) Neurturin evokes MAPK-dependent upregulation of ERG4 and KCC2 in developing neurons. Neural Plast., 2011, 1-8.

Maitra,M et al. (2010) Identification of GATA6 sequence variants in patients with congenital heart defects. Pediatr. Res., 68, 281-285.

McKenna,WL et al. (2011) Tbr1 and Fezf2 regulate alternate corticofugal neuronal identities during neocortical development. J. Neurosci., 31, 549-564.

Muñoz,EM et al. (2007) NeuroD1: developmental expression and regulated genes in the rodent pineal gland. J. Neurochem., 102, 887-899.

Otto,A., et al. (2010) A hypoplastic model of skeletal muscle development displaying reduced foetal muyblast cell numbers, increased oxidative myofibres and improved specific tension capacity. Dev. Biol. 343, 51-62.

Quentien,MH et al. (2006) Pituitary transcription factors: from congenital deficiencies to gene therapy. J. Neuroendocrinol., 18, 633-642.

Quinn,LM et al. (2000) The homeobox genes MSX2 and MOX2 are candidates for regulating epithelial-mesenchymal cell interactions in the human placenta. Placenta, 21, S50-S54.

Roberts,LD et al. (2011) The contrasting roles of PPARB and PPARG in regulating the metabolic switch between oxidation and storage of fats in white adipose tissue. Genome Biology, 12, R75.

Robertson,N.G. et al. (1991) Testis-specific expression of the human MYCL2 gene. Nucleic Acids Res., 19, 3129-3137.

Ropka-Molik,K, Eckert,R and PiÃ³wska,K (2011) The expression pattern of myogenic regulatory factors MyoD, Myf6 and Pax7 in postnatal porcine skeletal muscle. Gene Expr. Patterns, 11, 79-83.

Scariano,JK et al. (2008) Estrogen receptors alpha (ESR1) and beta (ESR2) are expressed in circulating human lymphocytes. J. Recept. Signal Transduct. Res., 28, 285-293.

Shaut,CAE et al. (2008) HOXA13 is essential for placental vascular patterning and labyrinth endothelial specification. PLoS Genet., 4.

Shen,L et al. (2010) Transcription factor HAND2 mutations in sporadic Chinese patients with congenital heart disease. Chin. Med. J. (Engl.), 123, 1623-1627.

Stephensen,C.B. (2007) Disruption of Rxra gene in thymocytes and T lymphocytes modestly alters lymphocyte frequencies, proliferation, survival and T helper type 1/type 2 balance. Immunology, 121, 484-498.

Sugitani,Y et al. (2002) Brn-1 and Brn-2 share crucial roles in the production and positioning of mouse neocortical neurons. Genes Dev., 16, 1760-1765.

Sun,Y et al. (2011) Phosphorylation state of Olig2 regulates proliferation of neural progenitors. Neuron, 69, 906-917.

Taylor,HS and Fei,X (2005) Emx2 reggulates mammalian reproduction by altering endometrial cell proliferation. Mol. Endocrinol., 19, 2839-2846.

Thomsen M.B., et al. (2009) Accessory Subunit KChIP2 Modulates the Cardiac L-Type Calcium Current, Circ. Res., 104, 1382-U1251.

Valenti,MT et al. (2008) Gene expression analysis in osteoblastic differentiation from peripheral blood mesenchimal stem cells. Bone, 43, 1084-1092.

Van der Flier,LG et al. (2009) Transcription factor achaete scute-like 2 controls intestinal stem cell fate. Cell, 136, 9003-912.

Vella,KR et al. (2007) Expression of the hypothalamic transcription factor Nhlh2 is dependent on energy availability. J. Neuroendocrinol., 19, 499-510.

Virard,I et al. (2006) Oligodendrocyte precursor cell generate pituicytes in vivo during neurohypophysis development. Glia, 53, 294-303.

Wang,C et al. (2011) Synergistic activation of cardiac genes by myocardin and Tbx5. PLoS One, 6, [Epub 2011, Aug 29].

Wilkinson,S.T. (2011) Partial plasma cell differentiation as a mechanism of lost major histocompatibility complex class II expression in diffuse large B-cell lymphyma. Blood, [Dec 13, Epub ahead of print].

Xiao,J et al. (2002) NYD-SP6, a novel gene potentially involved in regulating testicular development/spermatogenesis. Biochem. Biophys. Res. Commun., 291, 101-110.

Ye,DZ and Kaestner,KH (2009) Foxa1 and Foxa2 control the differentiation of goblet and enteroendocrine L- and D-cells in mice. Gastroenterology, 137, 2052-2062.

Yoshihara,SI et al. (2005) Arx homeobox gene is essential for development of mouse olfactory system. Development, 132, 751-762.

Zhang,P. Et al. (2000) PU.1 inhibits GATA-1 function and erythroid differentiation by blocking GATA-1 DNA binding. Blood, 96, 2641-2648.

Zhang,D et al. (2008) G-protein pathway suppressor 2 (GPS2) interacts with the regulatory factor X4 variant 3 (RFX4_v3) and functions as a transcriptional co-activator. J. Biol. Chem., 283, 8580-8590.

**Figure 1:** Tissue specific regulatory network of the porcine transcriptome with tissue specific transcription factors (TSTF) highlighted in red. Node size was mapped to average transcript abundance, node colour and shape were mapped to the different gene types: TS (grey squares), TF (grey triangles) and TSTF (red circles).


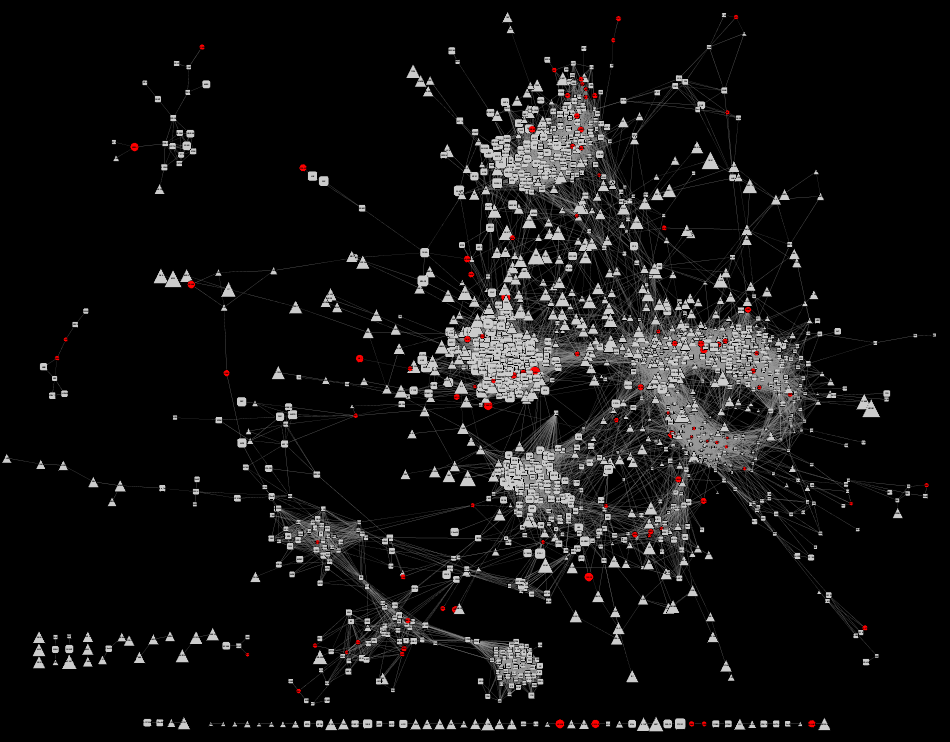

Supplement: Table S2 — Word document file listing the 112 tissue specific transcription factor genes, their expression and their location in the network. (DOC) [file pone.0046159.s004.doc]
